# Unusual, Uncommon, Intriguing, and Significant Causes of Kounis Syndrome: Important Medications and Chemicals Used to Treat Kounis Syndrome and Myocardial Infarction Can Cause Kounis Syndrome

**DOI:** 10.3390/jcdd12110423

**Published:** 2025-10-24

**Authors:** Nicholas G. Kounis, Cesare de Gregorio, Ming-Yow Hung, Grigorios Giamouzis, Marina A. Michalaki, Uğur Özkan, Alexandr Ceasovschih, Virginia Mplani, Periklis Dousdampanis, Sophia N. Kouni, Alexandros Stefanidis, Kassiani-Maria Nastouli, Maria Bozika, Nicholas Patsouras, Ioanna Koniari

**Affiliations:** 1 Department of Cardiology, University of Patras Medical School, Rio, 26500 Patras, Greece; kassienmarie@gmail.com (K.-M.N.); mariabozika29@gmail.com (M.B.); npatsouras@gmail.com (N.P.); iokoniari@yahoo.gr (I.K.); 2 Department of Clinical and Experimental Medicine, University of Messina Medical School, 98122 Messina, Italy; cesare.degregorio@unime.it; 3 Division of Cardiology, Department of Internal Medicine, Shuang Ho Hospital, Taipei Medical University, 18 No. 291, Zhongzheng Rd., Zhonghe District, New Taipei City 23561, Taiwan; myhung6@ms77.hinet.net; 4 Taipei Heart Institute, Taipei Medical University, Taipei City 110301, Taiwan; 5 Division of Cardiology, Department of Internal Medicine, School of Medicine, College of Medicine, Taipei Medical University, Taipei City 110301, Taiwan; 6 Department of Cardiology, University Hospital of Larissa, 41334 Larissa, Greece; grgiamouzis@gmail.com; 7 Department of Internal Medicine, Division of Endocrinology, University of Patras School of Health Sciences, Rio, 26500 Patras, Greece; mixmar@upatras.gr; 8 Department of Cardiology, School of Medicine, Trakya University, 22030 Edirne, Turkey; drugurozkan@hotmail.com; 9 Faculty of Medicine, Grigore T. Popa University of Medicine and Pharmacy, 700115 Iasi, Romania; alexandr.ceasovschih@yahoo.com; 10 Intensive Care Unit, Patras University Hospital, Rio, 26500 Patras, Greece; virginiamplani@yahoo.gr; 11 Department of Nephrology, Saint Andrews State General Hospital, 26221 Patras, Greece; dousdampanis@yahoo.gr; 12 Speech Therapy Practice, Queen Olgas Square, 26221 Patras, Greece; snkouni@yahoo.gr; 13 First Cardiology Department, General Hospital of Nikea, Agios Panteleimon Piraeus, 3 D. Mantouvalou Street, 18454 Piraeus, Greece; plato203@yahoo.com; 14 Department of Cardiology, Liverpool Center for Cardiovascular Science, Liverpool L14 3PE, UK

**Keywords:** adrenaline, aspirin, atropine, clopidogrel, heparin, corticosteroids, kisses, Kounis syndrome, leech, hirudotherapy, protamine sulfate

## Abstract

Mast cell degranulation and other interacting and linked cells, including T-lymphocytes, macrophages, eosinophils, and platelets, as well as a range of inflammatory mediators produced during an anaphylactic or allergic reaction, constitute the main causes of Kounis syndrome. Acute ischemia episodes, coronary spasm, atheromatous plaque erosion/rupture, and platelet activation can all be caused by histamine, tryptase, arachidonic acid derivatives, and chymase in the Kounis syndrome cascade. Kounis syndrome can be triggered by a variety of factors, including medications, hymenopteran stings, metals, foods, environmental exposures, illnesses, and immunizations. In addition, some unusual, rare, intriguing, and significant causes of Kounis syndrome have been discovered recently, namely the “kiss of death”, where human kissing and pet kissing can induce fatal Kounis syndrome. Moreover, the clinical conundrum is that several of the main drugs and substances used to treat myocardial infarction and Kounis syndrome, such as adrenaline (epinephrine), aspirin, atropine, clopidogrel, corticosteroids, heparins, protamine sulfate, and hirudotherapy can also initiate it. Therefore, physicians should be aware of this clinical discrepancy to prevent catastrophic consequences.

## 1. Introduction

The primary causes of Kounis syndrome include the degranulation of mast cells and other interacting and associated cells, such as T-lymphocytes, macrophages, eosinophils, and platelets, as well as a variety of inflammatory mediators generated during an anaphylactic or allergic reaction or attack. Histamine, tryptase, derivatives of arachidonic acid, and chymase can all contribute to acute ischemia events, coronary spasm, atheromatous plaque erosion/rupture, and platelet activation in the Kounis syndrome cascade. In addition to these well-established effects, new research indicates that mast cells influence both early-phase acute responses and late-phase chronic inflammation and tissue remodeling by releasing mediators that lead to microvascular spasm, endothelial dysfunction, and platelet-leukocyte aggregation formation [1]. Kounis syndrome is now recognized as a unique type of acute vascular syndrome that impacts the cerebral, mesenteric, peripheral, and venous systems in addition to the coronary arteries. Furthermore, Kounis syndrome is a multisystem and interdisciplinary illness rather than a single-organ vascular ailment. Drugs, hymenopteran stings, metals, foods, environmental exposures, diseases, and vaccinations might cause Kounis syndrome. Moreover, a number of peculiar, uncommon, fascinating, and important causes of Kounis syndrome have been identified in recent years, such as the “kiss of death”, in which kissing a person or pet kissing can cause deadly Kounis syndrome. In a paradoxical clinical scenario, Kounis syndrome might be induced by drugs and substances used to treat thrombosis, myocardial infarction, and Kounis syndrome. These include adrenaline (epinephrine), aspirin, atropine, clopidogrel, corticosteroids, heparins, protamine sulfate, and hirudotherapy.

## 2. Current Perspectives on Kounis Syndrome

The first classification of cardiovascular symptoms linked to anaphylactic, anaphylactoid, allergy, or hypersensitive responses as acute carditis, morphologic cardiac reactions, or rheumatic carditis of uncertain pathogenesis was based on blood pathology. In 1991, the allergic angina syndrome was first thoroughly characterized as a cardiac spasm [2]. Later known as Kounis syndrome, a condition characterized by endothelial dysfunction or microvascular angina leading to allergic acute myocardial infarction [3,4]. The primary causes of Kounis syndrome include a variety of inflammatory mediators generated post an anaphylactic or allergic reaction or insult from the degranulation of mast cells and other interacting and connected cells, such as T-lymphocytes, macrophages, eosinophils, and platelets. Histamine, tryptase, and arachidonic acid derivatives, as well as chymase, which functions as a converting enzyme, can all contribute to acute ischemia, coronary spasm, atheromatous plaque erosion/rupture, and platelet activation in the Kounis syndrome cascade. Drugs, hymenoptera stings, metals, foods, environmental exposures, medical disorders, and vaccinations consist all possible triggers of Kounis syndrome. According to recent studies, the incidence of this condition varies from 1.1% to 3.4% in individuals who have an allergic, hypersensitive, anaphylactic, or anaphylactoid insult. From 2007 to 2014, the National Inpatient Sample was used to compare baseline demographics and comorbidities with Kounis syndrome among patients who were mostly hospitalized for allergic, hypersensitive, or anaphylactic responses [5]. It can not only impact the coronary arteries, but also the mesenteric, cerebral, and peripheral arteries. Despite this, Kounis syndrome seems to be underdiagnosed. Initially, it was believed to be an uncommon ailment. The best way of diagnosis establishment and further treatment is to employ a high rate of suspicion [4].

**Figure 1:**
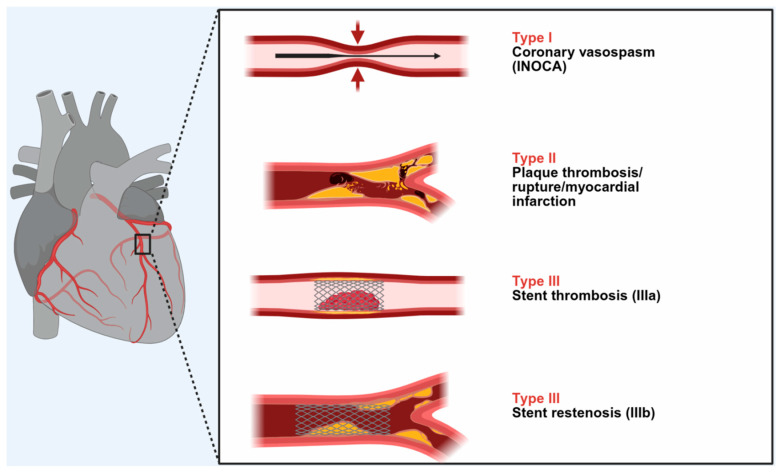
The types and subtypes of Kounis syndrome.

Three types of this condition have been identified thus far, as seen in Figure 1.

A myocardial infarction of type I or INOCA (Ischemia with No Obstructive Coronary Arteries) affects 76.6% of patients with normal or nearly normal coronary arteries, and it can be caused by histamine, chymase, or arachidonic acid products (leukotrienes, platelet-activating factor). Acute myocardial infarction with platelet activation and similar conditions that induce type I can also trigger type II, which affects 22.3% of patients with quiescent prior coronary disease. In total, 5.1% of patients may demonstrate type III stent thrombosis (subtype IIIa) or stent restenosis (subtype IIIb), which is caused by stent polymers, stent metals, eluted drugs, dual antiplatelets, and environmental exposures [6].

## 3. The Kiss of Death

### 3.1. Gereral Considerations

Kissing is an age-old method of expressing affection or simple erotic desire. “Kiss of death” is a phrase used to describe a behavior or relationship that has lethal or catastrophic outcomes. While the most well-known example is Judas’ “kiss,” who betrayed Jesus Christ in the Garden of Gethsemane sending him to his executioners, “kiss” may also serve as a mafia signal that someone has been marked for execution. In a variety of social contexts, including movies, sports, literature, music, technology, and even medicine, this type of expression is common. In the context of loving pets, “kissing” by insects and bugs refers to an offensive, defensive, feeding biting that has all negative effects. Licking and kissing are displays of love and devotion to their owners that can spread allergens and bacteria to people. Utilizing super-resolution microscopy to demonstrate how gold nanoparticles can effectively kill cancer cells by forming a crucial “golden” kiss of death on the nuclei and mitochondria is a metaphorical medical example of kissing [7]. In the real world, passionate kisses can sometimes have disastrous or even fatal outcomes. Unexpectedly deadly outcomes can arise from a friend’s passionate kiss, a pet’s tender kiss or licking, and a flying kisser bug’s hostile kiss. Kissing involves touching the saliva, skin, and oral mucosa, as well as inhaling substances. Consequently, it is anticipated that disease transmission may become feasible. Because of its flushing action, human saliva has a natural cleansing function. Nevertheless, human saliva can act as a vector for bacteria, viruses, and allergens, even though it contains antimicrobial defenses like antibodies and other antimicrobial proteins like lysozyme [8].

### 3.2. Human Kissing Inducing Allergy and Kounis Syndrome

**Figure 2:**
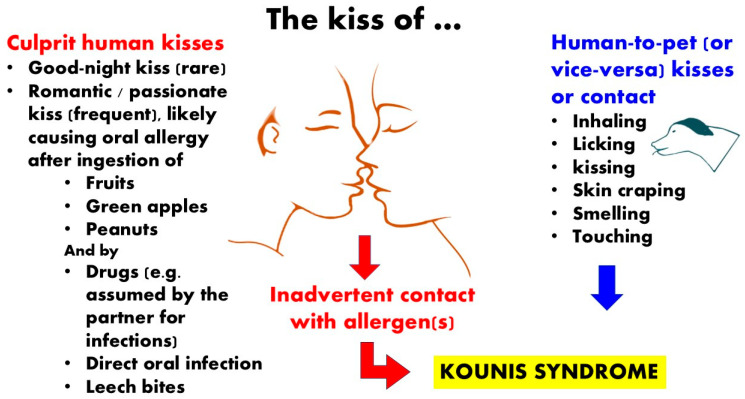
Green apples, shrimp, peanuts, and kiwifruits can cause allergies after a passionate kiss. Adorable dogs can act as “indirect hosts” that contaminate humans by licking, kissing, caressing, dandering, inhaling, or smelling.

There are reports of food allergens that can cause an allergic reaction when they are transferred from one person to another through physical contact, such as kissing. If one lover is sensitized to the food that the other just consumed, the close contact of two oral mucosae during kissing may result in an oral allergy syndrome. Some examples include the following: severe allergic reaction to a shellfish brought on by a good-night kiss [9], kiss-induced allergy to peanuts [10], oral allergy syndrome to green apples following a lover’s kiss [11], and kiwi fruit-induced oral allergy syndrome following a romantic kiss [12]. Kounis syndrome is an acute coronary syndrome linked to allergies, depicted in Figure 2, caused by eating actinidia chinensis [13]. In another case, a young man, age 23, who had previously experienced oral allergy syndrome to kiwifruit, suffered an acute myocardial infarction after a piece of kiwifruit consumption [14]. A second case of kiwifruit-induced Kounis syndrome has been documented in the literature since the first case [15]. A 2-year-old girl with a fish allergy developed facial urticaria and angioedema post her grandfather’s kiss after he had consumed fish two hours before [16]. On multiple occasions, a 45-year-old woman became sensitized to bacampicillin post her husband’s passionate kiss while he was taking antibiotics for gingivitis [17]. The skin, oral mucosa, and saliva can spread bacteria and viruses, in addition to allergens. One remarkable instance is the “kiss of death” of a 23-year-old South African man who, post oral contact with a woman with evidence of an active herpes simplex virus infection, developed fulminant hepatic failure and passed away from multiorgan failure as a result of overwhelming sepsis [16].

### 3.3. Pet Kissing Inducing Allergy and Kounis Syndrome

Humans may be impacted by the transmission of allergens and microbes through pet kissing, licking, and dander [18]. According to data from skin-prick tests, pet allergies may be the most prevalent perennial allergen in the Unted States, while small, suspended, particulate animal allergens may be present in 90% of all homes and the majority of public indoor spaces [19]. There are five other well-described allergens for both cats and dogs, even though Fel d 1 and Can f 1 are the most significant allergens for both. Car seats contain levels of dog and cat allergens that are significantly higher than the threshold levels for human sensitization and symptoms, and homes with pets have significantly higher levels of Fel d 1 or Can f 1 allergen compared to homes without pets [20]. Antibiotics are the primary treatment agents for microbial pet infections. In a report, 17 patients developed Kounis syndrome, which was confirmed by cardiac catheterization, positive skin tests for antibiotics, elevated IgEs, histamine, tryptase, and a positive leukocyte transformation test [21]. Beta-lactams were among the common causes of Kounis syndrome. The beta-lactam antibiotics were given intramuscularly, intravenously, and orally. All patients survived cardiac catheterization, even though type I and type II variants of this syndrome were found. Males accounted for 76% of the patients. In a somewhat intriguing study [22], an atopic patient who was sensitive to beta-lactams and had experienced two myocardial infarctions post oral amoxicillin administration experienced a third episode of Kounis syndrome-like acute myocardial infarction after being licked on the face by his devoted dog. Amoxicillin had previously been used to treat the dog. It has been discovered that when amoxicillin-containing saliva comes into contact with an atopic patient’s skin, it can further cause an allergic reaction and Kounis syndrome. Additionally, it is reported that, post oral administration of 750 mg of amoxicillin, sputum concentrations of the antibiotic are between 0.4 and 0.5 mg/L [23]. According to this report, sensitized people can develop Kounis syndrome without necessarily coming into contact with, breathing in, or consuming the responsible allergen [24]. By licking, kissing, touching, dandering, inhaling, or smelling, affectionate pets can serve as “indirect hosts” that can spread illness to people.

## 4. Clinical Paradox

Key medications and substances used to treat myocardial infarction and Kounis syndrome can cause Kounis syndrome.

### 4.1. Adrenaline (Epinephrine)

**Figure 3:**
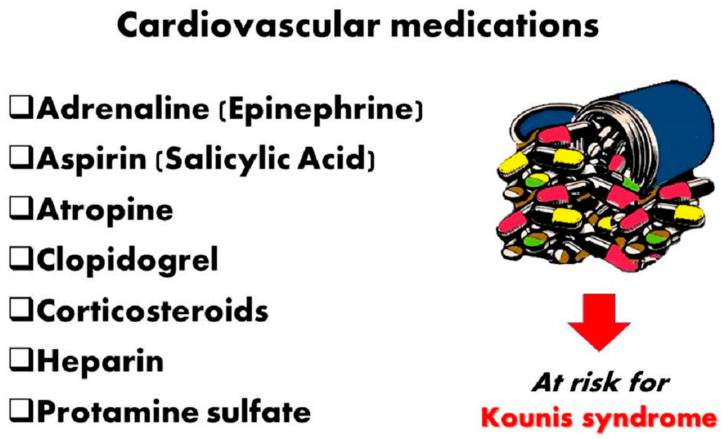
Kounis syndrome can be caused by important drugs used to treat myocardial infarction, creating a clinical dilemma.

Ironically, the medication that can save lives in cases of anaphylaxis, adrenaline, can also cause anaphylaxis on its own [25]. According to Drug Facts and Comparisons, a standard pharmacy reference published by Wolters Kluwer and updated monthly, sodium metabisulfite is a preservative found in all commercially available preparations of adrenaline [26]. A common antioxidant in the food and pharmaceutical industries is sodium metabisulfite. Metabisulfite, an additive agent of local anesthetics containing adrenaline, has been implicated in cases of anaphylactic shock that occurred during the administration of epidural anesthesia for caesarian sections. When sulfite-sensitive patients experience anaphylactic shock, this presents a therapeutic conundrum. This association should be known by medical professionals who treat anaphylactic shock. Thankfully, sulfite-sensitive patients can now receive free sulfite adrenaline from a commercial source (American Regent Inc., Pharmaceutical company, New Albany, OH, USA) [27]. Glucagon, which has been effectively used to treat anaphylaxis in patients on β-blockers, is a potential substitute in this case. Specifically, international guidelines recommend injecting exogenous adrenaline intramuscularly at a dose of 0.01 mg/kg of a 1:1000 (1 mg/mL) solution11, up to a maximum dose of 0.5 mg in adults. This procedure can save lives. Twelve Properly diluted solutions (1:10,000 [0.1 mg/mL] or 1:100,000 [0.01 mg/mL]) for intravenous administration may exacerbate coronary spasm. A challenging contemporary clinico-pharmacological combination is the Adrenaline, Takotsubo, Anaphylaxis, and Kounis (ATAK) complex [4]. “Attacking” is required to clarify its pathophysiology and etiology and to put preventative and therapeutic measures into action. Although adrenaline is the preferred medication for treating anaphylactic shock, its administration may cause coronary spasm Figure 3. Furthermore, Takotsubo syndrome can result from direct myocardial stunning that causes coronary spasm [28]. The mediators released during anaphylaxis can also cause Τakotsubo and coronary spasm [29]. Beyond the hemodynamic support during anaphylactic shock, adrenaline administration may raise plasma catecholamine levels, which would further support this vicious cycle, also linked with Kounis syndrome [30].

### 4.2. Aspirin

Over the past 30 years, basic and clinical research has provided insight into the role that thromboxane A2 (TXA2)-dependent platelet activation has in intestinal inflammation, atherothrombosis, primary hemostasis, tissue repair, and colorectal cancer. Low-dose aspirin is also used, and TXA2 biosynthesis is measured in both human and mouse models [31]. Despite this, the field of antiplatelets has advanced over these years, the use of aspirin plays a key role in the primary prevention of atherosclerotic cardiovascular disease. The focus has shifted from efficacy to safety, supporting aspirin-free antiplatelet regimens following percutaneous coronary intervention, and there have been multiple attempts to create antiplatelet medications that are safer and more effective than aspirin. It is well established that low-dose aspirin can not only prevent atherosclerotic cardiovascular disease, but can also prevent colorectal (and other digestive tract) cancer by acting as a chemopreventive agent [32]. Aspirin can cause Kounis syndrome, despite its positive effects on cardiovascular conditions. Kounis syndrome, also referred to as the Samter–Beer triad, is a combination of nasal polyps, asthma, and aspirin allergy that causes vasospasm and myocardial infarction. In order to identify the Kounis syndrome quickly and focus treatment on reducing the allergic reaction, all doctors should be aware of this special clinical entity [33]. Another report describes a case of Kounis syndrome secondary to asthma triggered by taking aspirin, which was meant to treat angina pectoris [34]. Furthermore, a case study of a patient with a history of aspirin allergy who developed coronary vasospasm post aspirin consumption is presented in another paper [35].

### 4.3. Atropine

Atropine can be used to treat a number of conditions, such as anticholinergic poisoning, pupil dilatation, and symptomatic bradycardia without reversible causes. The alkaloid atropine was first produced by synthesizing it from Atropa belladonna. Just l-hyoscyamine is pharmacologically active out of the racemic mixture of d- and l-hyoscyamine. There are only combination products that contain oral atropine. Atropine inhibits the effects of acetylcholine and other choline esters by acting as a competitive, reversible antagonist of muscarinic receptors. Usually found as a sulfate salt, atropine can be given intravenously, subcutaneously, intramuscularly, intraosseously, endotracheally, or ophthalmically. Even though severe allergic reactions to atropine are extremely uncommon, patients who have experienced an allergic reaction in the past are more likely to experience a severe reaction in the future. We believe that the sulfate salt for intravenous use may be the cause of the allergic reaction leading to Kounis syndrome. Free sulfate salt preparations of atropine might need to be developed. The following two reports of atropine-induced Kounis syndrome have been already published: The authors of the first report [36] express the opinion that this is the first pediatric instance of Kounis syndrome caused by intravenous atropine. Silent myocardial ischemia can cause coronary vasospasm, which can lead to sudden death. A variety of pharmacological agents are among the numerous precipitant factors, concluding that acute coronary syndromes linked to anaphylactic or anaphylactoid reactions characterize Kounis syndrome. In a different study [37], the authors stated that atropine is rarely associated with allergic reactions. However, a case of a 25-year-old male patient with a history of persistent bradycardia, psychotic disorder, and cannabis dependent syndrome was demonstrated who experienced urticarial rash, dyspnea, and chest pain post atropine administration. The electrocardiogram displayed ST segment alterations that subsided following symptom relief. Coronary arteries were found to be normal by coronary angiography. Despite being rarely reported, it is a serious condition that treating physicians find difficult to diagnose, even though it may mimic ST segment elevation myocardial infarction.

### 4.4. Clopidogrel

Aspirin and more modern antiplatelet drugs are employed in antiplatelet therapy, as platelet aggregation is a biological target for the treatment of thromboembolism and other clotting disorders. These agents include the following: clopidogrel, prasugrel, and ticagrelol. Clinical professionals treating patients need to be well-versed on the pharmacology, pharmacokinetics, pharmacodynamics, clinical effectiveness, and safety of routinely used antiplatelet agents [38]. The most typical symptom of a clopidogrel allergy is a rash. It is critical to differentiate this from other causes of rash in patients who recently received a coronary stent. The majority of clopidogrel hypersensitivity reactions can be effectively treated with antihistamines and short-term oral corticosteroids, but some persistent reactions may necessitate clopidogrel discontinuation. It has long been practiced to substitute an alternative thienopyridine, such as ticlopidine, when clopidogrel discontinuation is necessary. However, according to a recent study, there may be a 27% chance that non-life-threatening allergic reactions, which are typically comparable to those occurred with clopidogrel, will recur in these patients [39]. Three reports of clopidrogrel-induced Kounis syndrome have been published so far. In the first report [40], a 61-year-old man was hospitalized due to increasing chest pain in the context of recurrent episodes of excruciating chest pain during normal activities and at rest and heavy smoking. In addition, he had previously experienced allergic reactions, atopic eczema, and hypertension. The final diagnosis was Type I Kounis syndrome secondary to an allergic reaction to clopidogrel. Another report described a 56-year-old male patient with Kounis Syndrome who experienced angioedema, respiratory distress, and vasospasm in the right coronary artery following a loading dose of clopidogrel [41]. Moreover, recurrent acute stent thrombosis associated with clopidogrel-induced allergic reaction leading to Kounis syndrome was reported in a 44-year-old male patient [42]. Desensitization to clopidogrel has become necessary as a result of all of these incidents. For clopidogrel-sensitive individuals who need long-term dual antiplatelet medication, desensitization is safe and very successful [43].

### 4.5. Corticosteroids

Although corticosteroids are frequently used to treat allergic responses, they can also cause anaphylaxis with Kounis syndrome and acute, delayed, local, or systemic allergic reactions [44]. By inhibiting phospholipase A2 and eicosanoid production, they can prevent the release of arachidonic acid from mast cell membranes. In addition to mediating the manufacture of annexin or lipocortin, which are chemicals that regulate inflammatory cell activation, adhesion molecule expression, and transmigratory and phagocytic processes, corticosteroids can also induce cell death. Among the implicated reasons are hapten production, antigen–antibody interaction, and drug pollutants. The following pathways allow for systemic corticosteroids to cause allergic reactions [45]: (1) There have been reports of anaphylactic reactions to methylprednisolone involving IgE antibodies. (2) The medications that most commonly cause type 1 (immediate) adverse responses are succinate esters of hydrocortisone and methylprednisolone. (3) Hapten synthesis is aided and functions as a full antigen because succinate esters have a stronger affinity for serum proteins and a greater solubility in water. (4) The medications that most commonly cause type 1 (immediate) adverse responses include methylprednisolone and hydrocortisone succinate esters.

A patient with polymalformative syndrome who had previously undergone gastrostomy for necrotizing enterocolitis and experienced anaphylaxis to cow’s milk proteins experienced multiple episodes of anaphylaxis, including urticaria, eyelid edema, laryngospasm, and severe dyspnea, just a few minutes after receiving 10 mg of methylprednisolone sodium succinate intravenously [45]. Moreover, after receiving methylprednisolone succinate pulse treatment for neuromyelitis optica and systemic lupus erythematosus, two patients presented anaphylactic shock with cutaneous and systemic symptoms [46]. Another 52-year-old woman who had previously demonstrated an allergy to anti-haemorrhoid lotions and ointments experienced a widespread symmetrical pruritic eruption after receiving 1 milliliter of triamcinolone acetonide intra-articularly [47]. Atopic diathesis patients are especially at risk. Before administering any specific medicine, including corticosteroids, a full and comprehensive history of drug reactions or allergies is required.

### 4.6. Heparines

Heparin is a blood anticoagulant that makes antithrombin more active. Antithrombin inactivates its physiological target enzymes, Thrombin, Factor Xa, and factor IXa. The clinical paradox is that physicians are cautious of the potential for bleeding side effects when they prescribe heparins, since they might occasionally cause thrombocytopenia, which can worsen bleeding. However, thrombocytopenia may result in unanticipated severe thrombosis. Indeed, the primary antigen that activates platelets via the Fc gamma receptor II (FcγRII) and causes thrombosis is the three-component immunological complex, made up of heparin, platelet factor 4 (PF4), and Immunoglobulin G (IgG). The ensuing extensive thrombosis increases platelet consumption and worsens thrombocytopenia.

**Figure 4:**
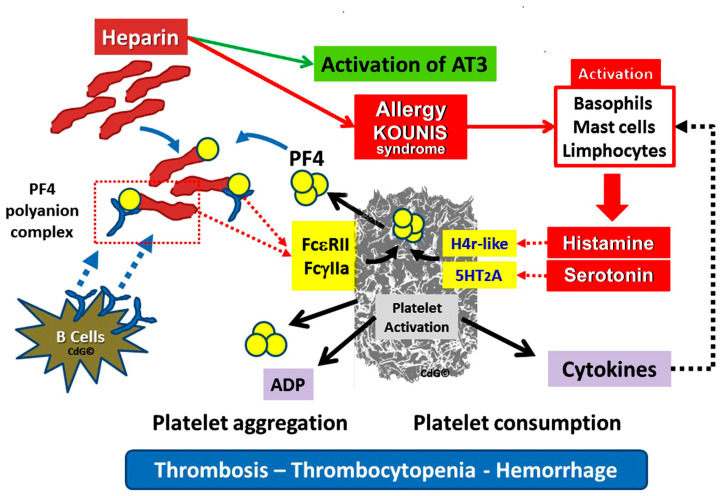
Simplified mechanism(s) underlying heparin-induced thrombocytopenia (HIT) syndrome, a paradox of platelet thrombosis with thrombocytopenia and hemorrhage.

Heparin-induced thrombocytopenia (HIT) is characterized by a decrease in platelet counts and a hypercoagulable condition [48]. Significant morbidity and death are linked to thromboembolic problems in patients presenting with HIT, as depicted in Figure 4. Given the widespread use of heparin for line flushes, heparin-coated catheters, and the prevention and treatment of thromboembolism, this represents a substantial burden [49].

Heparin can rarely cause Kounis syndrome, as in the following 5-days-of-age patient: Kounis Syndrome was suspected in a full-term newborn who had a Rashkind-atrioseptostomy and stenting, and had intermittent episodes of acute coronary syndrome. Tests on the skin of nickel and titanium yielded no results. The suspected medications were tested using basophil activation test and lymphocyte transformation (LTT). A heparin-positive LTT and non-response to basophils were found using the BAT (Basophil Activation Test). After a fresh heparin dose was inadvertently administered, new acute coronary syndrome was developed. According to the authors, this was the first case of Kounis syndrome in an infant triggered by heparin therapy [50]. Moreover, heparin treatment for a 67-year-old lady with deep vein thrombosis in her lower limbs resulted in acute thrombus development and ST-elevation myocardial infarction. Subsequent heparin-PF4 IgG antibody and serotonin release assay were both positive, supporting the diagnosis of HIT [51]. Therefore, the clinical conundrum is that heparin used to treat thrombotic events such as Kounis syndrome can also induce thrombosis and Kounis syndrome.

### 4.7. Protamine Sulfate

Protamine sulfate is a drug used to counteract heparin’s effects. It is used primarily to counteract the effects of heparin after delivery, cardiac surgery, and plethora operations, as well as to treat heparin overdose and low-molecular-weight heparin overdose. It is administered intravenously, and its effects usually start to show up within five minutes. Hypersensitivity responses, vomiting, low blood pressure, and a sluggish heartbeat are typical adverse effects. Anaphylaxis is one of the severe allergic responses. For patients who have undergone a vasectomy, the risk is higher. The fifth pregnancy-related use has not been thoroughly investigated, despite the fact that there is no proof of any negative effects. Protamine functions by binding itself to heparin. For many years, protamine has been utilized to speed up the removal of the sheaths while still in the lab following catheter ablation and percutaneous coronary interventions. It has been demonstrated to speed up walking without raising the risk of thrombosis or problems with the access site. Hypersensitivity responses may result in pulmonary edema, dermatitis, hypotension, and, in rare cases, Kounis syndrome. There have been several reports of Kounis syndrome linked to the use of protamine sulfate. Although protamine sulfate shock is prevalent, Kounis syndrome might be concealed within it [52,53]. Therefore, such situations shouldn’t be handled as a straightforward protamine shock. The mechanism of protamine shock and associated risk factors, as well as the pathophysiology and type of Kounis syndrome in patients who had protamine shock, should be highlighted in these publications [54]. Furthermore, one should consider Kounis syndrome while considering the importance of ST-elevation during anaphylactic shock [55].

## 5. Leech Is Used to Remove Thrombosis but It Can Induce Kounis Syndrome and Thrombosis

Since bloodletting was a widespread custom in ancient Greece, leeches (βδελα-λες, vthela-les in Greek) have been used medicinally for thousands of years. At the time, medical professionals thought that drawing blood from a patient could both prevent and treat illness. Leeches were used more frequently for bloodletting than rudimentary tools. One must first comprehend the paradigm of disease from Hippocrates’ (~460–370 BC) time 2300 years ago in order to understand the justification for bloodletting. According to Hippocrates, four fundamental elements of life—earth, air, fire, and water—were analogous to the four basic humors of humans: blood, phlegm, black bile, and yellow bile. Each humor was associated with a specific personality type (sanguine, phlegmatic, melancholic, and choleric) and was centered in a specific organ (brain, lung, spleen, and gall bladder) [56]. Although leech therapy has proven to be effective, there is ongoing debate regarding its safety and potential side effects [57]. Hirudotherapy (leech therapy) made a comeback in the 1970s, where the medicinal leech, Hirudomedicinalis, became the most widely used leech species for a variety of medical purposes, especially in complementary medicine for ailments like osteoarthritis and microsurgery for post-operative venous congestion. Hirudotherapy can be a useful adjunctive treatment; however, in order to achieve the greatest outcomes, careful consideration of both positive and negative factors must be made. Leech saliva contains physiologically active compounds, including hirudin, which has anticoagulant, anti-inflammatory, and vasodilating effects. Recently, a hirudin-based fusion protein prodrug combined with microneedles for long-term antithrombotic treatment has been successful in achieving continuous protection, on-demand antithrombotic bioactivity recovery, and a streamlined dosage schedule [58]. However, despite the anticoagulant action, six cases were found in a review of the published literature on leech allergies, five of which included mild allergic reactions like pruritus [59]. Moreover, despite having no previous history of allergies, a 58-year-old man had anaphylaxis after being bitten by a leech, which resulted in myocardial infarction [60]. Two cases documented the onset of Kounis syndrome [61].

## 6. Perspectives

Allergy responses can result from a variety of drugs, situations, exposures, and chemical compounds. However, there are several unusual, uncommon, fascinating, and important causes of Kounis syndrome. Several drugs and substances that are used to treat thrombosis and Kounis syndrome have the potential to cause both the condition and anaphylactic responses. Atropine is often administered intravenously as a sulfate salt. Metabisulfite is a substance that is added to local anesthetics that include adrenaline. Protamine sulfate is used to reverse the effects of heparin. Sulfate is among the most often used drugs in clinical practice. On rare occasions, it might result in Kounis syndrome and anaphylactic reactions. Therefore, it is advisable to consider this response before drug administration. Moreover, those who are susceptible to allergic myocardial infarction and allergic angina can be protected by reducing their immunoglobulin E (IgE) levels. This can explain the reason why not every patient who has an allergic reaction and who also does not have Kounis syndrome seems to be addressed by the following: their blood’s IgE levels are lower. There may be hope for reducing allergy-associated Kounis syndrome by focusing on the IgE route and the inflammatory processes linked to it [62].

## Data Availability

No new data were created or analyzed in this study. Data sharing is not applicable to this article.
